# How Single Molecule Real-Time Sequencing and Haplotype Phasing Have Enabled Reference-Grade Diploid Genome Assembly of Wine Grapes

**DOI:** 10.3389/fpls.2017.00826

**Published:** 2017-05-17

**Authors:** Andrea Minio, Jerry Lin, Brandon S. Gaut, Dario Cantu

**Affiliations:** ^1^Department of Viticulture and Enology, University of California, DavisDavis, CA, United States; ^2^Department of Ecology and Evolutionary Biology, University of California, IrvineIrvine, CA, United States

**Keywords:** heterozygosity, inbreeding depression, cabernet sauvignon, comparative genomics, grape pan-genome

## High heterozygosity is a challenge for grape genome assembly

Domesticated grapevines (*Vitis vinifera*) have relatively small genomes of about 500 Mb (Lodhi and Reisch, 1995; Jaillon et al., 2007; Velasco et al., 2007), which is similar to other small-genomes species like rice (430 Mb; Goff et al., 2002), medicago (500 Mb; Tang et al., 2014), and poplar (465 Mb; Tuskan et al., 2006). Despite their small genome size, the sequencing and assembling of grapevine genomes is difficult because of high levels of heterozygosity. The high heterozygosity in domesticated grapes may be due, in part, to their domestication from an obligately outcrossing, dioecious wild progenitor. Domesticated grapes can be selfed, in theory, because their mating system transitioned to hermaphroditic, self-fertile flowers during domestication. In practice, however, selfed progeny tend to be non-viable, presumably due to a high deleterious recessive load and resulting inbreeding depression. As a consequence of these fitness effects, most grape cultivars are crosses between distantly related parents (Strefeler et al., 1992; Ohmi et al., 1993; Bowers and Meredith, 1997; Sefc et al., 1998; Lopes et al., 1999; Di Gaspero et al., 2005; Tapia et al., 2007; Ibáñez et al., 2009; Cipriani et al., 2010; Myles et al., 2011; Lacombe et al., 2013).

One such cultivar is Cabernet Sauvignon, one of the most widely cultivated wine grape cultivars. Cabernet Sauvignon was produced from a cross between Sauvignon Blanc and Cabernet Franc sometime before the seventeenth century in the Aquitaine region of France (Bowers and Meredith, 1997). Whether a spontaneous hybrid or a product of human breeding, all of the Cabernet Sauvignon grown around the world is thought to have resulted from this single hybridization event. Just as the parents of Cabernet Sauvignon have been identified, the genetic origin of many other important wine grape cultivars is known, and they often originate from the direct crossing of common, distantly-related cultivars (Strefeler et al., 1992; Ohmi et al., 1993; Qu et al., 1996; Bowers and Meredith, 1997; Sefc et al., 1998; Lopes et al., 1999; Crespan and Milani, 2001; Vouillamoz et al., 2003, 2004; Di Gaspero et al., 2005; Vouillamoz and Grando, 2006; Lacombe et al., 2007, 2013; Tapia et al., 2007; Boursiquot et al., 2009; Ibáñez et al., 2009; Cipriani et al., 2010; Myles et al., 2011; García-Muñoz et al., 2012). Due to this intraspecific hybridization process, levels of heterozygosity in grape cultivars can easily exceed 11% (Jaillon et al., 2007).

**Figure 1:**
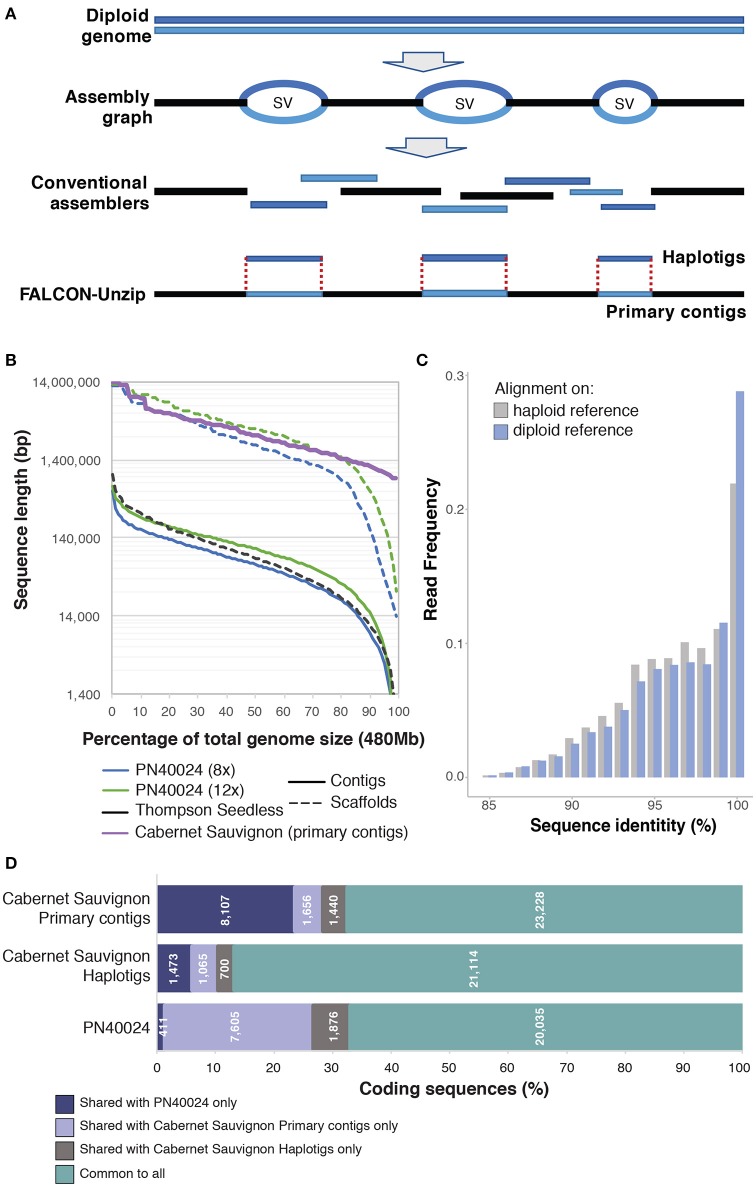
**Comparison of conventional assemblers and their application to the grapevine genome using FALCON-Unzip and its results on Cabernet Sauvignon. (A)** A diagram comparing how conventional assemblers and FALCON-Unzip resolve homozygous and heterozygous regions of diploid genomes. **(B)** Comparison of sequence length distribution between the primary contigs of Cabernet Sauvignon assembled with FALCON-Unzip and other *Vitis vinifera* genome contig and scaffold assemblies. **(C)** Distribution of sequence identity between RNAseq reads and reference when mapping is done only on primary contigs or on a combination of primary contigs and haplotigs. **(D)** Shared coding genes sequences between Cabernet Sauvignon haplotypes and PN40024. Predicted coding sequences from the Cabernet Sauvignon primary contigs were aligned using GMAP (Wu and Watanabe, 2005) to the Cabernet Sauvignon haplotigs and the PN40024 chromosomes to identify the shared part of the represented gene space. Only alignments with identity ≥80% and coverage ≥66% were considered. In similar fashion, coding sequences from the Cabernet Sauvignon haplotigs were aligned against the primary contigs and the PN40024 chromosomes, and coding sequences from PN40024 were aligned against both primary contigs and haplotigs of Cabernet Sauvignon.

High heterozygosity is challenging for genome assembly, because heterozygous genomes typically produce more fragmented sequences than haploid or homozygous genomes of similar size and complexity (Yu et al., 2005; Argout et al., 2011; The Tomato Genome Consortium, 2012). The goal of standard assembly approaches is to collapse homologous regions with sufficient similarity into haploid consensus sequences, but divergent haplotypes in heterozygous regions typically result in multiple, difficult to resolve assembly paths which must then be assembled separately. Additionally, the boundaries between haploid consensus contigs and heterozygous regions cannot be resolved with a unique path; as a result they are left unlinked, which breaks assembly contiguity (Figure 1A). Altogether, elevated heterozygosity increases fragmentation and inflates the size of the total assembly, potentially doubling the genome size if the majority of the two homologous genomes are assembled separately (Huang et al., 2012; Li et al., 2012; Safonova et al., 2015). Fragmentation and retention of redundant regions can also lead to inaccurate gene models, apparent paralogous genes and duplicated blocks, incorrect gene copy number, and synteny breaks.

## Initial attempts to sequence the grape genome

Despite the challenges in assembling heterozygous genomes, the commercial and cultural importance of the grapevine has led to several sequencing attempts. Two genome reference drafts for the common grapevine were released in 2007 (Jaillon et al., 2007; Velasco et al., 2007). Remarkably, these were the first genomes of any fruiting crop to be sequenced and only the fourth for flowering plants. These reference genomes, both of which utilized the Pinot Noir cultivar, were assembled using different approaches to address heterozygosity. The first genome by Jaillon et al. reduced heterozygosity by inbreeding a line of Pinot Noir (var. PN40024) to ~7% heterozygosity (Jaillon et al., 2007). To produce the second genome, Velasco et al. sequenced a Pinot Noir clone (ENTAV115) directly then assembled contigs that represented separate homologous chromosomes (Velasco et al., 2007). Unsurprisingly, these early efforts are poor by current standards. The PN40024 genome had ~8.4-fold coverage and was assembled into 19,577 contigs with a contig N_50_ of only 65.9 kbp. Later sequencing increased coverage to up to 12x and the contig N_50_ of the PN40024 genome to 102.7 kb (Figure 1B). The ENTAV115 genome used both Sanger paired-reads and 454 sequencing to achieve a total coverage of ~4.2x. Although riddled with gaps and potentially omitting large regions of repetitive sequences where genes could be located, the two genomes provided valuable insights into grape genomes. Together they revealed that the Pinot Noir genome features: (i) ~30,000 protein-coding genes, comparable to Arabidopsis but about 75% of rice and poplar; (ii) a high proportion of repetitive elements comprising an estimated ~40% of the genome; (iii) complex patterns of gene duplications consistent with one or more paleopolypoidy events; (iv) expansion of gene families that influence the organoleptic properties of the berry; (v) a typical number (~200) of NBS-LRR genes, which often function in disease resistance, and (vi) a standard complement of genes involved in disease signaling pathways. Despite its limitations, the PN40024 genome assembly has proven to be invaluable to the grape research community. Cited in over 2,000 articles, it has served as a reference in more than 3,000 genome-wide transcriptional analyses.

Following the publication of the PN40024 genome in 2007, no genome reference of equivalent or greater quality has been released for *V. vinifera*. Only a handful of studies have attempted to use *bona fide* genome-wide approaches to measure diversity within the species (Giannuzzi et al., 2011; Da Silva et al., 2013; Di Genova et al., 2014; Cardone et al., 2016). With the advent of second generation short read sequencing, attempts were made to perform *de novo* assembly and reference based resequencing of grape cultivars. These attempts failed to provide a high quality representation of the sequenced grape genotypes. A *de novo* approach was adopted to assemble the genome sequence of Thompson Seedless, a ubiquitous multipurpose cultivar. Despite an enormous sequencing depth (327x), the short fragment size did not permit resolution of repetitive regions, resulting in an extremely fragmented assembly (Di Genova et al., 2014; Figure 1B). For the wine grape cultivar Tannat (Da Silva et al., 2013), the authors applied a reference based assembly approach, which had proved to be effective in assembling multiple Arabidopsis genotypes (Gan et al., 2011). However, reference-based assembly failed to reconstruct genotype specific sequences with Tannat data, demonstrating that large scale resequencing initiatives like the 1,000 Human Genome project (Auton et al., 2015) and the 1,001 Arabidopsis Genomes project (Alonso-Blanco et al., 2016) would not succeed for *Vitis*. In fact, while the approach supported variant calling with *de novo* assembly to resolve regions highly divergent in sequence between Tannat and PN40024, it was unable to recover regions absent in the reference but present in Tannat. Consequently, over 10% of the gene space was not represented in the assembly, illustrating that the genomic sequence of one cultivar is insufficient for representing the total variability of the species. To improve representation of the *V. vinifera* pan-genome and encompass the variability of the species, we need the complete *de novo* assembled genomes of additional genotypes. Moreover, as grape cultivars are intraspecific hybrids of different genotypes, assembly of each genome should include a diploid representation of the genome to preserve information about the characteristics of each haplotype.

## Recent developments in grape genome sequencing

Single Molecule Real Time (SMRT) DNA sequencing (Pacific Biosciences) has emerged as a leading technology for characterizing complex structural variations, supporting and refining the assembly of complex genomes in hybrid fashion or alone for reconstructing highly continuous assemblies of both small and highly repetitive genomes (Chin et al., 2013; Doi et al., 2014; Huddleston et al., 2014, 2016; Gordon et al., 2016; Ricker et al., 2016; Seo et al., 2016; Vij et al., 2016). The advantage of SMRT technology arises from the delivery of long reads, currently averaging over 30 kbp and potentially approaching 100 kbp. In addition to facilitating assembly of more contiguous genomes, long reads carry the necessary information to phase haplotypes over multiple kilobase distances. The open-source software, FALCON-unzip (Chin et al., 2016), was developed specifically to utilize the long reads generated using SMRT sequencing technology and assemble diploid genomes into highly contiguous and correctly phased diploid genomes. The algorithm first constructs a string graph composed of “haploid consensus” contigs together with bubbles representing structural variant sites between homologous loci. Sequenced reads are then phased and separated for each haplotype on the basis of heterozygous positions. Phased reads are finally used to assemble the backbone sequence (primary contigs) and the alternative haplotype sequences (haplotigs) (Figure 1A). The combination of primary contigs and haplotigs constitute the final diploid assembly with phased single-nucleotide polymorphisms and structural variants between the two haplotypes.

We have recently reported the assembly using SMRT technology and FALCON-unzip of the highly heterozygous diploid genome of Cabernet Sauvignon (Chin et al., 2016), one of the most widely cultivated wine grape cultivars. As it is the progeny of Cabernet Franc and Sauvignon Blanc, two cultivars with extremely divergent phenotypical traits, reconstructing the diploid structure of Cabernet Sauvignon is necessary for identifying the alleles inherited from the parent cultivars. We sequenced the Cabernet Sauvignon genome with a coverage depth of ~140x using SMRT sequencing technology. Sequencing reads were then assembled using FALCON-unzip into a highly contiguous genome that integrated phased haplotype information. FALCON-unzip generated a set of primary contigs (591.4 Mbp in 718 contigs with N_50_ = 2.17 Mbp, Figure 1B) that covers one of the two haplotypes, and a set of correlated haplotigs (367.8 Mbp in 2,037 contigs with N_50_ = 0.80 Mbp). The assembled sequences exceed PN40024 contigs and Thompson Seedless scaffolds by nearly two orders of magnitude in size (Figure 1B), ranking this assembly not only as the best *V. vinifera* genome assembly but also among the highest quality plant genomes published to date, including other genomes sequenced with SMRT technology (Sakai et al., 2015; VanBuren et al., 2015; Jiao et al., 2016; The UC Davis Coffee Genome Project, 2017). Symptomatic of the extreme divergence in allele sequences in *Vitis*, the length of the primary assembly was inflated with respect to the expected genome size, illustrating one of the challenges of sequencing highly heterozygous genomes (Chin et al., 2016). After manual removal of un-phased haplotigs, the primary assembly is an ideal candidate for scaffolding or hybrid assembly with optical maps to produce a genome assembly of even higher quality.

Preliminary gene model prediction identified over 34,000 protein coding sequences on the primary assembly of the Cabernet Sauvignon genome and nearly 24,000 on the haplotigs (Chin et al., 2016). Just a few hundred of PN40024 annotated coding genes did not find any suitable alignment on the Cabernet Sauvignon assembly (411 genes; identity ≥80% and coverage ≥66%), but nearly 4,900 Cabernet Sauvignon loci could not be found on the PN40024 genome (Figure 1D). These results are in accordance with other studies that reported presence/absence polymorphisms of gene models between wine grape cultivars (Da Silva et al., 2013; Venturini et al., 2013; Jiao et al., 2015), but the high number of genes not found in PN40024 likely reflects its incompleteness. Moreover, nearly 2,100 coding sequences identified in the Cabernet Sauvignon haplotigs were not found on the primary assembly (Figure 1D). While limited by the preliminary status of the annotation, these observations point to a high degree of structural variation between homologous chromosomes. Moreover, these structural variations are likely to have functional consequences since they encompass coding sequences. The variability between haplotypes may also impact and potentially confound the analysis of RNAseq data. In the worst case, the expression of haplotype-specific loci that are not represented on the reference genome would be assigned to the most similar genomic region of the reference, which is likely to generate expression mismeasurement artifacts. As shown in Figure 1C, in the presence of a diploid reference (primary contigs plus haplotigs), about 10% more RNAseq reads map at ≥99% identity. This observation suggests that when both alleles are represented in the reference reads align to their respective haplotype; RNAseq can therefore be used to determine allelic specific gene expression.

## Conclusions

Genome resequencing projects of both prokaryotic and eukaryotic organisms have clearly shown that one genome sequence is insufficient to properly describe the genetic characteristics of a species (Tettelin et al., 2005; Donati et al., 2010). In order to grasp comprehensive genetic variability and complete gene pools in outcrossing species, such as grape, we also need to go beyond the generation of haploid consensus sequences and focus our efforts to begin assembling diploid genome sequences with phased haplotypes. As discussed in this article, long read sequences and bioinformatic tools that take advantage of them have solved a critical bottleneck in grape genomics. As long-range scaffolding technologies, such as those based on proximity ligation–based methods like Hi-C (Putnam et al., 2016) or optical maps (Hastie et al., 2013; Yoon et al., 2016) are optimized for highly heterozygous plant genomes, we expect that reference-grade genome references will quickly become available for many grape species and cultivars of interest. This genomic information will allow us to identify core sequences that are common to all cultivars, as well as dispensable sequences comprising partially shared and non-shared genes that contribute to inter-cultivar phenotypic variation. This genomic information will also enable the identification of the genetic bases of economically important traits to accelerate the breeding of new cultivars and rootstocks.

## Author contributions

DC and AM conceived the article. Figure was prepared by AM and DC. AM, JL, BG, and DC wrote the first draft of the manuscript. DC revised and finalized.

## Conflict of interest statement

The authors declare that the research was conducted in the absence of any commercial or financial relationships that could be construed as a potential conflict of interest.
